# The Role of Nutrition in Maintaining the Health and Physical Condition of Sports Volunteers

**DOI:** 10.3390/nu16193336

**Published:** 2024-10-01

**Authors:** Mateusz Rozmiarek

**Affiliations:** Department of Sports Tourism, Faculty of Physical Culture Sciences, Poznan University of Physical Education, 61-871 Poznan, Poland; rozmiarek@awf.poznan.pl

**Keywords:** volunteering, sport, nutrition, volunteer management, dietary habits, health, physical conditions, energy intake, diet, nutritional education

## Abstract

Nutrition plays a key role in maintaining health and physical condition, particularly for active individuals, including athletes. It can therefore be assumed that individuals performing physically demanding tasks during the organization of sporting events, such as volunteers, should also pay attention to their nutrition. While the importance of diet for athletes has been widely studied, the impact of nutrition on sports volunteers remains under-researched. Volunteers often have to cope with varying degrees of physical and mental exertion, which may affect their nutritional needs. A qualitative study was conducted using in-depth individual interviews (IDIs) with 17 sports volunteers who had experience in organizing various sporting events. Participants were purposefully selected based on specific inclusion criteria, which included active involvement in sports volunteering (with a minimum of two years of experience in volunteer activities) as well as volunteering experience at sports events of various scales. The interviews aimed to understand the eating habits, dietary awareness, and impact of nutrition on health and physical fitness. The data were transcribed and subjected to thematic analysis, focusing on coding responses and identifying recurring themes. Most participants did not place much importance on their diet, making random food choices due to a busy lifestyle and lack of time. Only a few volunteers consciously adjusted their diet when they had knowledge of the physically demanding tasks they were expected to perform during their volunteer work. The majority of volunteers relied on less reliable sources of nutritional information, such as blogs or social media, rather than credible sources of knowledge. This study revealed that many individuals involved in sports volunteering are unaware of the impact of diet on their fitness and health. There is a need for nutritional education for this group to improve their awareness of the importance of a balanced diet in the context of increased physical activity. It is also advisable to provide better nutritional conditions during sporting events and to promote the use of professional sources of information about healthy eating.

## 1. Introduction

Nutrition plays a key role in maintaining health as well as optimal physical and mental condition, especially among physically active individuals [1]. Athletes, in particular, pay close attention to dietary aspects, adjusting their nutrition to meet their training and competition needs to maximize performance and improve results [2]. However, the importance of proper nutrition is not limited to those directly involved in sports. Individuals supporting the organization of sporting events, such as organizers, technical staff, or sports volunteers, may also face significant physical and mental demands due to their responsibilities [3].

Volunteers involved in organizing sporting events form a specific group that not only plays essential roles in the management of sports events but also engages in various physical activities related to their duties [4]. These may include tasks requiring appropriate levels of strength, endurance, agility, or fitness, such as setting up courses, assisting with the arrangement of sports equipment, or performing other organizational duties that may be challenging for the average person. Therefore, a balanced diet may also be crucial for sports volunteers to maintain high energy levels, focus, and the ability to perform tasks effectively, which ultimately contributes to the quality and efficiency of sports event organization. Proper nutrition in this group can support not only their health but also contribute to the success and smooth execution of events.

Most of the existing research on the impact of diet on physical condition or performance focuses on professional athletes [5,6], which limits the perspective on other, equally important social groups. While professional athletes represent a group with specific dietary needs, sports volunteers, who are required to maintain an adequate level of physical fitness, remain largely overlooked in scientific studies. To date, there is a lack of systematic analyses of their eating habits and dietary needs, even though their lifestyle may require distinct dietary recommendations.

Sports volunteers working in conditions that demand significant physical activity will have increased energy and nutrient requirements in terms of their diet. Work during sports events such as marathons, triathlons, or track and field competitions often involves long hours of intense activity, presenting them with unique nutritional challenges. Proper dietary adjustments are crucial to maintain both physical health and fitness during their volunteer duties. Similar to athletes, their increased caloric needs resulting from physical activity should be met with a diet rich in macronutrients such as carbohydrates, proteins, and fats to meet the body’s energy and recovery needs [7]. It is also important to emphasize that adequate hydration and supplementation with certain micronutrients, such as iron or vitamins, can play a key role in optimizing physical performance [8].

However, not all volunteers are expected to maintain high levels of physical fitness, as some of them fill roles that require more organizational or administrative skills. Examples of such roles include managing participant registrations, overseeing race offices, distributing starter kits, supporting VIP guests, or working in media and communication tasks. In these cases, the ability to work in a team and precision and information management skills are essential, rather than physical fitness. For these volunteers, adhering to general healthy eating principles is fundamental, as confirmed by studies on the differing nutritional needs depending on the level of physical activity [9].

The nutritional needs of sports volunteers, like those of athletes, can vary depending on individual metabolic factors such as basal metabolic rate, body composition, or tolerance to specific food components. Personalizing their diet could therefore be a crucial factor and a significant challenge for this group [10], although for many sports event organizers, this may seem unrealistic, as volunteers are often viewed as auxiliary staff. However, according to scientific research, the eating habits of individuals performing physically demanding tasks can change depending on the intensity of their activity and the roles they assume [11]. Volunteers who dedicate more time to organizing and participating in sports events may be more aware of their dietary needs and strive to better adjust their eating habits to meet their energy demands, even during the preparation stages of their duties. On the other hand, those less involved in sports volunteering may not always realize the importance of optimizing their diet.

Additionally, stress and fatigue resulting from long hours of volunteering can negatively affect food choices, further complicating the maintenance of proper fitness and health levels [12,13]. Another important issue is what volunteers eat, or if they eat at all, during sports events. Proper nutrition directly impacts their well-being, especially given the physical demands of their roles. However, appropriate food options are not always available to them during such events. Volunteers may have limited access to balanced meals, leading them to opt for quick, highly processed foods with low nutritional value, which negatively affects their health and performance [14]. For this reason, ensuring appropriate nutritional conditions during such events should be a priority to support both the health and efficiency of volunteers, regardless of other factors [15].

This study aimed to analyze the daily dietary choices of participants, their understanding of the significance of diet for health and physical fitness—an aspect that is crucial for many volunteer roles—and to identify the sources of nutritional knowledge influencing their dietary approach. The following research hypotheses were formulated:

**Hypothesis** **1.** *Most sports volunteers do not attach significant importance to their daily diet, resulting in random food choices that are not aligned with their energy needs, given the intense nature of their volunteer activities.*

**Hypothesis** **2.** *Sports volunteers who do not pay attention to their diet and rely on random meals exhibit lower physical fitness during sports events compared to those who consciously plan their meals according to their energy requirements.*

**Hypothesis** **3.** *Sports volunteers use various sources of nutritional information, with professional sources being less frequently utilized compared to less reliable sources, such as blogs or social media.*

The results of this study will provide a better understanding of how dietary habits affect the health and physical fitness of sports volunteers and how the level of dietary awareness and sources of information impact their well-being. The final conclusions could form the basis for developing recommendations to improve the diet and health of individuals involved in organizing sports events.

## 2. Materials and Methods

This study was qualitative in nature and employed in-depth individual interviews (IDIs) as the primary research method [16]. Fontana and Frey [17] described in-depth individual interviews as one of the best ways to understand people and explore topics in depth. The individual approach to respondents, compared to, for example, moderated or unmoderated focus groups, leads to results containing more details, as well as greater engagement in discussing sensitive topics or those related to private life [18].

The choice of this research method was driven by the specific nature of this study, which generated a need for obtaining in-depth, complex information about eating habits, dietary awareness, and the perception of the impact of nutrition on physical condition by sports volunteers. The qualitative approach allowed for flexibility in the investigation and the adaptation of questions to the individual experiences of the participants [19], enabling a deeper understanding of their motivations, challenges, and barriers related to nutrition.

### 2.1. Sample Selection

Seventeen sports volunteers with experience in various types of sports events held over the past two years were invited to participate in this study. All study participants were involved in the 2023 European Games and were recruited from the pool of volunteers from this event. In our study, we applied specific criteria to standardize the attributes of the volunteers. Participants were intentionally selected based on precise inclusion criteria, which included active involvement in sports volunteering, requiring a minimum of two years of experience and participation in sports events of various scales. Additionally, we considered factors related to having a similar number of male and female participants (9 women and 8 men) as well as diversity in terms of education and occupation (for those who were not students or pupils). This approach aimed to ensure group homogeneity and minimize potential bias, resulting in reliable findings. The participants engaged in assisting various sports disciplines and performed different volunteer roles, which allowed for a better understanding of the specific nutritional requirements arising from diverse tasks. Table 1 presents an anonymized list of the study participants.

### 2.2. Justification for Sample Size

The sample size (n = 17) was determined according to the principles of qualitative research, where the number of participants is often guided by the point of data saturation. Data saturation means that no new, significant themes or information emerge from further interviews that could impact the understanding of the research topic. In this study, saturation was reached after conducting 17 interviews, confirming that this sample was sufficient to obtain comprehensive data. This sample size is also supported by numerous scientific publications dedicated to qualitative research methodology [20,21].

### 2.3. Procedure

This study was conducted using semi-structured interviews, which allowed for open-ended questions and provided respondents with the freedom to express themselves. This approach facilitated the collection of more nuanced responses and the discovery of themes that may not have been initially anticipated in the research protocol. The interviews focused on three main areas: the volunteers’ daily eating habits, their nutritional awareness regarding meal planning, and their sources of nutritional information. This method provided a comprehensive view of the group’s dietary behaviors and diverse approaches to healthy eating and its significance while performing volunteer roles at sports events.

To avoid research bias or variation in the questions asked, all interviews were conducted by one person—the author of this study. Interviews with volunteers were conducted from 1 July to 15 August 2023. Each interview lasted approximately one and a half hours, although the topics discussed were part of a broader exploration of dietary issues in the context of sports volunteering. Interviews were recorded with the participants’ consent.

The interviews were carried out in accordance with research ethics standards. Before the interviews began, participants were informed about the purpose of the study, its nature, and the voluntary nature of their participation. Each participant gave informed consent to participate in the study and to the recording of the interview. They were also assured of the anonymity and confidentiality of the collected data. The results of this study were presented in a manner that does not allow for the identification of individual participants.

### 2.4. Data Analysis

The interview data were subjected to thematic analysis in four stages: (1) transcription of data and preliminary reading of the materials; (2) coding of participants’ responses and identification of recurring themes; (3) grouping of codes into larger thematic categories; (4) interpretation of results and drawing conclusions based on identified themes. Each stage of the analysis was conducted with adherence to the standards of qualitative research rigor, which enhanced the credibility and validity of the data interpretation.

In order to ensure greater transparency and rigor in the analysis, an iterative and multi-stage approach to thematic analysis was applied. After the initial transcription of the interviews, the transcripts were read multiple times to fully understand the participants’ responses. The data was coded on two levels: (1) open coding, which identified potentially relevant themes, and (2) axial coding, which grouped thematic connections and enabled the identification of key motifs. This process was supported by analytical tools such as NVivo, ensuring systematic coding.

All codes were then compared and grouped to refine thematic categories. The results of the analysis were confronted with the limited available studies in related thematic areas, which allowed for embedding the findings in a broader context. The researcher applied a rigorous approach to coding and categorizing the data. To minimize subjectivity and enhance the objectivity of the results, the researcher repeatedly verified the identified themes and compared them with the initial codes to ensure consistency in interpretation.

## 3. Results

This study was conducted with a group of 17 sports volunteers involved in organizing various sports events, including distance runs, triathlons, and track and field competitions. These volunteers performed a range of roles, from logistical organization to direct support for participants, requiring varying levels of physical activity. This study involved interviews about their dietary habits, dietary changes based on their volunteer activities, and sources of nutritional information. Below are the main findings from these interviews, illustrated with quotes from the respondents.

### 3.1. Daily Dietary Habits of Sports Volunteers

In the studied group of volunteers, the vast majority (n = 14) did not pay much attention to their daily diet. Their dietary choices were mostly random. One participant noted “Honestly, I eat whatever is at hand—I work intensely, and when you’re on the go all day, you don’t pay attention to what you eat” (V8). Another volunteer added “Diet isn’t something I think about daily. I live with my parents, so I eat whatever is available at home” (V4). Another participant admitted, “Even if I wanted to eat healthier, I often just don’t have the time. I study, work, and meet friends in my free time, so eating takes a back seat” (V5).

However, a few individuals attempted to plan their meals, although they often settled for whatever was available at the moment. For example, one participant said “I try to plan my meals, but I don’t always succeed. When I’m away from home, I sometimes end up at McDonald’s, but it’s better to eat something than nothing” (V6). Another added “I don’t have time to regularly think about what I eat, but I try to eat healthily when I can” (V3). Another volunteer mentioned “Sometimes I end up eating in a rush, but when I can, I choose fruits or make sandwiches myself” (V10).

Only three volunteers (n = 3) demonstrated full dietary awareness and made an effort to tailor their nutrition to their body’s needs. One of them stated “Regular consumption of vegetables is crucial for me. Since I started paying more attention to my diet, I feel much better” (V11). Another volunteer added, “I make sure to drink plenty of water and avoid processed foods. I’ve noticed that a healthy diet not only improves my physical fitness but also my concentration” (V13). Meanwhile, another volunteer remarked “I eat regularly because I know how important proper nutrition is for recovery after an intense day” (V1).

### 3.2. Nutritional Awareness and Meal Planning among Sports Volunteers

In this study, the majority of participants (n = 14) did not place significant importance on their diet, both in terms of the quality of their meals and their alignment with the intensity and type of sports events they were involved in. These volunteers often relied on the available meals provided by the event organizers, eating randomly without much planning, and did not consider the energy needs associated with more demanding roles. One participant noted, “I always eat whatever is served on site. Often it’s sandwiches or some snacks, but I don’t pay attention to whether it’s good for me” (V2). Another respondent mentioned, “During long events, I’m often forced to eat whatever is available, even if it’s not the healthiest. You just need to eat something to have the energy” (V12). Another participant said, “Most of the time we eat what is offered to us, if there’s even something planned. Sometimes the organizer only gave me an apple for the whole day, and I had to make do with that, though it was very tough” (V14).

Most of these individuals did not perceive the connection between diet and physical condition during their volunteer work. Their eating habits were largely random, and healthy eating was not a priority. As one volunteer noted, “I don’t really worry about what I eat. Most of the time, I’m busy, so I eat whatever is available without much thought” (V16). Another respondent added, “My diet is very random. If there’s nothing to eat and there’s a kebab or some fast-food restaurant nearby, that’s what I eat. Healthy food isn’t a priority for me, especially during intense volunteer days” (V15).

Only three respondents (n = 3) demonstrated a conscious approach to their diet, adjusting their eating habits to match the intensity of their volunteer work. These individuals paid attention to their energy needs, particularly for physically demanding roles. One volunteer observed, “When I’m involved from early morning setting up the running course and placing barriers, I know I need to eat something substantial before and after. I always choose oatmeal for breakfast and then an energy bar during the event” (V1). Another participant added, “During events like triathlons, I always carry bananas and electrolyte water with me. I need to stay well-hydrated and replenish energy because it’s really long events” (V13). These volunteers highlighted that diet plays a crucial role in maintaining their physical condition and health during long volunteer days. One of them noted, “If I don’t eat a proper meal before the event, I get tired quickly. I know that good nutrition is essential to survive the whole day on my feet, and the organizer doesn’t always provide decent food” (V11). For these individuals, meal planning and including healthy snacks, such as nuts, fruits, or yogurt, was an important part of their preparation for engaging in sports volunteering.

### 3.3. Sources of Nutritional Knowledge among Sports Volunteers

Volunteers obtained their nutritional knowledge from various sources. A small portion of the volunteers (n = 3) used professional and reliable sources, such as scientific articles, specialized nutrition websites, athlete guidelines, or even consultations with dietitians. As one participant noted, “I read a lot about nutrition, especially in the context of sports, as I try to run recreationally three times a week. I’ve also consulted with a dietitian a few times in my life because I know that what I eat directly affects how I feel” (V1). Another participant added, “I regularly read blogs run by experts in healthy eating. I believe that well-balanced meals are important for maintaining high fitness levels” (V13). Notably, this trio was the oldest in the group, which might suggest that as people age, their awareness and interest in professional sources of nutritional information increase. Over time, life experiences and the need to maintain good health may lead to a greater inclination to use reliable sources of knowledge, contributing to their greater attention to a balanced diet.

Among the remaining volunteers (n = 14), most admitted they did not actively seek nutritional information and relied mainly on intuition and available options. One participant said, “I don’t specifically look for information about food. I eat what I have, and I don’t think about it” (V4). Another added, “I don’t worry too much about what I eat. I usually eat whatever is at hand without checking if it’s healthy” (V9). There were also volunteers who mentioned occasionally using more popular sources of nutritional information, such as blogs, social media, and advice from friends. They often referred to using tips available online through popular search engines. As one volunteer mentioned, “I look for information on Instagram or articles that pop up in Google. I try to choose reliable sources, but I admit that sometimes I go with what friends suggest” (V10). Another volunteer added, “I don’t always pay attention to where the food information comes from. I often just browse what’s easily available online” (V17). Another participant noted, “I’ve read blogs or general advice about healthy eating, but I’m not sure how reliable those sources were” (V7).

## 4. Discussion

The conducted research revealed significant relationships between the level of nutritional awareness among sports volunteers and their dietary habits and perception of the impact of diet on physical condition. The results indicate some concerning trends with important implications for both understanding the needs of volunteers and for potential interventions aimed at improving their health and effectiveness during sports events.

### 4.1. Awareness of Diet and Dietary Habits

The results of this study revealed significant gaps in nutritional awareness among sports volunteers, which may have serious consequences for their health and physical condition. Most respondents did not pay attention to the quality of their diet, consistent with previous research highlighting widespread issues with meal planning even among individuals engaged in intense physical activities [22]. The literature emphasizes that poor nutrition, such as frequent consumption of fast food, can lead to health problems, including increased risk of obesity and metabolic diseases [23,24].

The findings also show that volunteers often eat randomly, relying on available options during events, which may lead to deficiencies in key nutrients. In the context of sports, inadequate nutrition can affect performance, as confirmed by Manore and colleagues [25]. Deficiencies in protein, carbohydrates, and micronutrients can lead to muscle weakness, slowed recovery, and overall deterioration in physical performance [26]. Similar effects may therefore affect volunteers engaged in physically demanding roles during sports events, who are also at risk of potential nutritional deficiencies due to poorly balanced diets.

Interestingly, even those volunteers who attempt to plan their meals were not always able to adjust their diet to the dynamic conditions of sports events, suggesting a need for better nutritional organization in such situations. Optimal meal planning, especially before prolonged and intense activities, is crucial for maintaining adequate energy levels and effectiveness [27]. Research indicates that improving meal planning and increasing nutritional awareness can significantly impact health and overall well-being [2].

### 4.2. The Significance of Diet in the Context of Health and Physical Condition

The results of this study suggest that the vast majority of volunteers are not fully aware of the impact of diet on health and physical condition, and as such, they do not adjust their dietary habits to the specific physical demands of sports volunteering. These practices are inconsistent with the findings of Hawley et al. [27], who emphasized the importance of diet periodization, meaning adjusting calorie, macronutrient, and micronutrient intake to the specific energy needs of an activity. Our results revealed a lack of understanding of the role diet plays in maintaining physical condition and health, a point already established by Logue et al. [28] in the context of active athletes. Our participants often viewed food merely as a means to satisfy hunger, rather than a crucial element for their physical performance. These findings suggest a need for education and increased awareness within this group to enable better alignment of their diet with their body’s needs.

On the other hand, individuals with a higher level of dietary awareness reported difficulties in maintaining consistent dietary habits. Similar issues were described in studies by Boyle et al. [29], who found that physically active individuals often encounter barriers such as lack of time, access to appropriate food, and logistical challenges, leading to irregular and often haphazard meal consumption. Participants from this group clearly indicated that their dietary choices were situation-dependent, which could lead to suboptimal health outcomes in the long term.

### 4.3. Lack of Knowledge and Intervention Opportunities

The results of this study reveal significant gaps in nutritional knowledge among sports volunteers. These findings align with Barbee’s observations [30], who noted that many people rely on unverified sources, which can lead to the adoption of incorrect nutritional advice. Our research shows that the majority of volunteers use unreliable sources of nutritional information, such as social media or lifestyle articles. The increased availability of information on the internet, including blogs and social media, contributes to the spread of unverified nutritional information [31]. Consequently, this can negatively impact the quality of dietary decisions and the effectiveness of dietary strategies.

One of the key challenges is therefore to provide appropriate sources, resources, and tools for sports volunteers so that they can make more informed nutritional decisions. In this context, it is crucial to implement specific strategies and interventions. For example, sports event organizers could provide pre-event nutrition briefings that give volunteers information on optimal nutrition and how to adjust their diet to their level of activity. Additionally, it is important to ensure that healthy meal options are available at event locations. Such measures can support volunteers in maintaining an appropriate level of energy and physical condition, which in turn enhances the overall quality of event organization.

Moreover, research by Sánchez-Díaz et al. [32] indicates that a low level or complete lack of nutrition education often leads to suboptimal dietary decisions that can impair physical condition. Although these studies pertain to professional athletes, their findings can also be applied to sports volunteers.

Lack of nutritional knowledge and reliance on unverified sources among sports volunteers not only limits their ability to optimally support their health and physical condition but may also contribute to long-term health issues. As highlighted by Devlin and Belski [33], low levels of knowledge about basic nutrition principles affect the ability to properly adjust the diet to the intensity of physical tasks. As a result, meeting increased energy needs becomes extremely challenging, which can lead to chronic fatigue, nutrient deficiencies, and issues with recovery after exertion. Spronk et al. [34] note that a lack of access to professional nutritional advice among physically active individuals, which can also be related to volunteers, often results in decisions based on popular myths and trends that lack scientific support. Thus, implementing educational intervention programs is crucial to increasing awareness about healthy eating and its impact on physical performance. In the context of sports volunteering, such knowledge could translate into improvements in both the quality of volunteer work and their health, minimizing the risk of injuries and exhaustion.

### 4.4. Strengths and Limitations

A strong point of the conducted study was the use of individual interviews, which allowed for a deep exploration of participants’ personal experiences and motivations, providing multidimensional and rich data. This approach made it possible to understand the dynamics between nutritional knowledge and dietary choices, as well as their impact on health and physical condition, which is crucial in research on dietary habits. Additionally, the analysis covered volunteers’ dietary awareness, enabling the identification of differences in approach between various participant groups and the identification of barriers to practicing healthy eating. The specific context of this study—volunteers involved in sports events, characterized by a high level of physical activity—makes it unique and provides valuable data on the impact of diet on physical performance in this group, which has not been extensively studied from this perspective before.

However, this study has certain limitations that may affect the generalization of the results. First, this study was conducted in one country, Poland, which may limit the applicability of the findings to a broader population of sports volunteers. Further research with a larger and more diverse participant group is needed to better understand the influence of factors such as cultural differences, education level, and socioeconomic environment on dietary habits. Additionally, the use of interviews may lead to subjectivity in responses, especially regarding the self-assessment of dietary habits. In the future, it would be worthwhile considering supplementing this study with quantitative methods, such as dietary diaries or anthropometric measurements, which could provide more objective data. Quantitative methods allow for more precise and less participant-biased results, as well as enable the analysis of a larger amount of data, increasing the reliability of conclusions. This approach also facilitates the identification of trends and correlations that may be invisible in qualitative data. Their use can therefore significantly enhance the quality of research, leading to more accurate and universal conclusions, which are particularly important in the context of future studies on population health and dietary habits.

## 5. Conclusions

The results of this study show that the dietary habits of sports volunteers are varied, but in most cases, they are not given much attention. The majority of participants consumed random meals, often relying on available, highly processed products. Only a few respondents demonstrated full awareness of their diet and made an effort to adjust their nutrition to the physical demands of volunteering. These results suggest a need for greater education on healthy eating, especially in the context of increased physical exertion and the challenges associated with working during sports events. Further research should also take into account the differences in the nutritional needs of volunteers depending on the type of sports event and the cultural contexts in which these events occur, in order to better tailor dietary recommendations to variable conditions. 

These conclusions also highlight the importance of providing appropriate meals during sports events. Volunteers who do not have access to a balanced diet may experience increased fatigue and decreased effectiveness, which can negatively impact the organization of events. Therefore, event organizers should ensure conditions that support healthy eating, which would not only benefit the volunteers’ health but also enhance the quality of their work and contribute to the success of the events being organized.

## Figures and Tables

**Table 1:** Table of participants.

Alias	Gender	Age	Education Level	Occupation
V1	Male	25	Master’s	Teacher
V2	Male	19	Secondary	Student
V3	Female	20	Secondary	Student
V4	Female	18	Primary	Pupil
V5	Male	22	Bachelor’s	Tour guide
V6	Male	20	Secondary	Student
V7	Female	18	Primary	Pupil
V8	Male	24	Vocational secondary	Electrician
V9	Male	21	Bachelor’s	Paramedic
V10	Female	23	Master’s	Sociologist
V11	Male	28	Master’s	Insurance agent
V12	Female	19	Secondary	Student
V13	Female	25	Master’s	Psychologist
V14	Female	18	Primary	Pupil
V15	Male	23	Master’s	Corporate employee
V16	Female	19	Secondary	Student
V17	Female	20	Secondary	Casual worker

## Data Availability

The original contributions presented in the study are included in the article, further inquiries can be directed to the corresponding author.
